# Disseminated *Talaromyces marneffei* infection associated with haemophagocytic syndrome in an HIV-negative patient in northern China: a case report

**DOI:** 10.1186/s12879-023-08953-y

**Published:** 2024-01-08

**Authors:** Hui Yang, Ming Liu, Nannan Xu, Lulu Yang, Sai Wen, Shanshan Wang, Chunmei Qu, Ke Xu, Enhua Sun, Wen Cui, Hui Liu, Gang Wang

**Affiliations:** 1 Department of Infectious Disease, Qilu Hospital, Cheeloo College of Medicine, Shandong University, 250012 Jinan, Shandong China; 2 Jinan Center for Disease Control and Prevention, 250021 Jinan, Shandong China; 3 Microbiology Laboratory, Qilu Hospital, Cheeloo College of Medicine, Shandong University, 250012 Jinan, Shandong China

**Keywords:** *Talaromyces marneffei*, Nonendemic areas, HIV-negative patient, Case report

## Abstract

**Background:**

*Talaromyces marneffei* is endemic to eastern India, Southeast Asia, and Guangdong and Guangxi provinces in China. It is common in immunocompromised individuals, especially in HIV-infected patients.

**Case presentation:**

A 66-year-old male who had a history of hypertension and resided in Shandong Province (Northern China) was admitted for recurrent fever for one month. The patient had recurrent fever, multiple lymphadenopathies, hepatosplenomegaly, a back rash, and a progressive decrease in white blood cells and platelets. *Talaromyces marneffei* was isolated from peripheral blood and bone marrow after admission, and suspected fungal cells were found via lymph node pathology. The patient’s infection secondary to haemophagocytic syndrome continued to worsen despite antifungal, anti-inflammatory, and symptomatic treatment, leading to death due to multiple-organ failure.

**Conclusion:**

Although rare, infection due to *Talaromyces marneffei* in HIV-negative patients has been increasing in recent years, and we should be vigilant about “new” infections in nonendemic areas.

## Background

Talaromycosis marneffei is an opportunistic mycosis caused by the thermal dimorphic fungus pathogen *Talaromyces marneffei*. It is endemic to eastern India, Southeast Asia, Guangdong Province and Guangxi Province in China and has been reported in other provinces as the floating population number has increased annually [1–5]. In 2013, Hu Y et al. reviewed 668 cases of *Talaromyces marneffei* infection in mainland China, with 99.4% of the cases being in southern China [3]. From the geographical distribution map of *Talaromyces marneffei* infection in 2019 created by Cao CW et al., we found that most cases were reported in the southern part of China, including Guangdong Province, Guangxi Province, Yunnan Province and Fujian Province. No cases were reported in Shandong Province [6]. Talaromycosis marneffei is common in immunocompromised individuals, especially in HIV-infected patients [7]. However, infection due to *Talaromyces marneffei* in HIV-negative patients has been increasing in recent years with the use of immunosuppressants, corticosteroids, and oncological chemoradiotherapy [8, 9].

*Talaromyces marneffei* mainly invades the reticuloendothelial system of human monocytes and macrophages and is characterized by local infection or disseminated infection [10]. Local infections are rare and involve infection of a single organ, such as the lung, leading to local symptoms. Disseminated infection can involve multiple systems and organs throughout the body and can manifest as fever, anaemia, weight loss, fatigue, hepatosplenic lymph node enlargement, cough, expectoration and gastrointestinal discomfort. Some patients may present with central nervous system involvement [5, 6, 8, 11]. The clinical manifestations of *Talaromyces marneffei* are not typical, and this disease is not easy to identify early. Once disseminated infection occurs, the disease progresses rapidly and can be life-threatening if antifungal treatment is not administered in a timely manner.

We report a case of *Talaromyces marneffei* infection in a patient in Shandong Province (northern China) with no evidence of immunodeficiency to increase awareness of *Talaromyces marneffei* infection in nonendemic areas.

## Case presentation

**Fig. 1:**
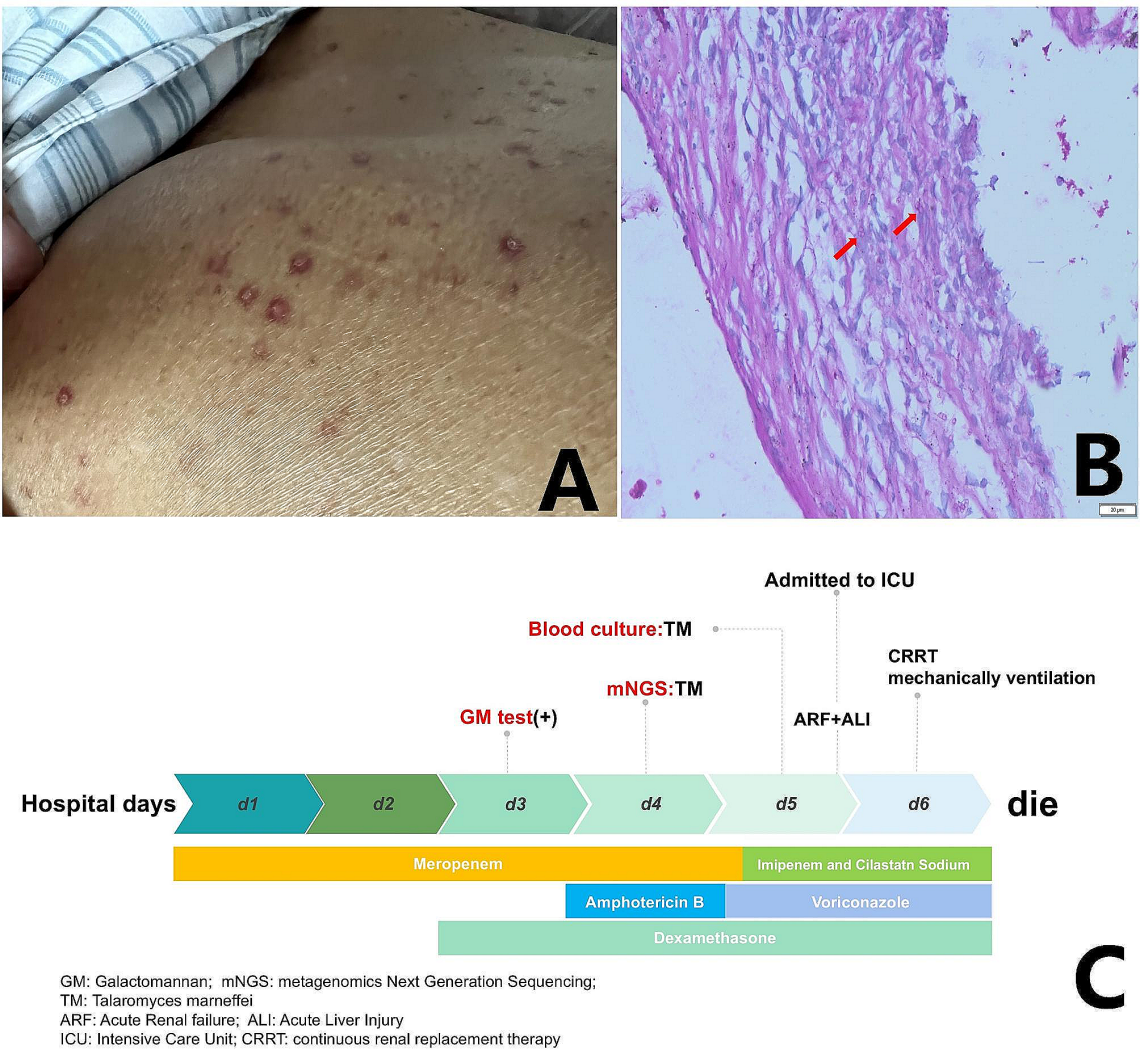
(**A**) showed scattered red rashes on the back; (**B**) The lymph node pathology specimen was restained and suspected fungal cells were found; (**C**) Hospitalization course with the timeline of pathogen and treatment

A 66-year-old male school worker with a history of hypertension who resided in Shandong Province, which is located in northern China, was admitted to the local hospital on April 2022 because of a 30-day history of recurrent fever (Tm 39.0 °C), chills, sore throat, fatigue, cough and weight loss. Laboratory results showed normal haemoglobin, platelets, renal function and electrolytes but elevated leukocyte (13.53 × 10^9^/L) with 83.40% neutrophils, C-reactive protein (CRP 71.88 mg/L) and procalcitonin (PCT 0.723 ng/ml) and slightly elevated alanine aminotransferase (133U/L) and aspartate aminotransferase (152U/L). Chest computed tomography (CT) revealed chronic bronchitis and multiple enlarged lymph nodes in the bilateral hilum of the lung, mediastinum and supraclavicular region, and suggesting lymphoma. The mediastinal lymph node obtained by bronchoscopy was sent for histological examination, and pathological examination indicated necrotic and inflammatory fibrous connective tissues and several atypical cells. He was treated with cephalosporins and ibuprofen, but his fever persisted, with his temperature fluctuating between 37.5 and 39.6 °C. On May 10, laboratory results showed normal leukocyte counts (4.19 × 10^9^/L), haemoglobin and platelet counts but highly elevated CRP levels (73.72 mg/L) and neutrophil ratios (91.60%). On May 16, the patient was admitted to our hospital for systematic diagnosis and treatment. Upon admission to the hospital, physical examination revealed poor physical condition and a body temperature of 38.9 °C, and he still experienced cough, expectoration, and chest distress. Dermatological examination revealed scattered red rashes on his back (Fig. 1A), and superficial lymph nodes were not palpable. Due to the unknown cause of fever, we made a cautious differential diagnosis and actively investigated related causes such as infection, rheumatic and immune diseases, and tumor diseases. Laboratory results showed significantly decreased leukocytes (1.35 × 10^9^/L) and platelets (29 × 10^9^/L) but highly elevated CRP (178.77 mg/L), PCT (32.03 ng/ml), triglycerides (5.86 mmol/L), ferritin (> 40,000 ng/ml), alanine aminotransferase (358U/L) and aspartate aminotransferase (1435U/L). Cytokine detection revealed highly elevated IL-6 (659.54 pg/ml), IL-8 (6514.34 pg/ml) and IL-10 (333.92 pg/ml) levels. His haemoglobin, fibrinogen, renal function and electrolytes were within the normal range. A fungal test revealed normal 1-3-β-D-glucan but elevated galactomannan. Blood culture yielded *Talaromyces marneffei*, and bone marrow culture confirmed that *Talaromyces marneffei* was present.

*Talaromyces marneffei* infection has rarely been reported in northern China. To verify the culture results, we sequenced peripheral blood samples to confirm the infection. The sequencing results revealed *Talaromyces marneffei*, with 3,965,512 sequence reads and 96.55% genome coverage. Pathology of the lymph node specimen was restained, and suspected fungal cells were found (Fig. 1B). This patient had recurrent fever, multiple lymphadenopathies, splenomegaly, weight loss, cough, sputum, and rash, and blood and bone marrow cultures were positive. Disseminated *Talaromyces marneffei* infection was confirmed [3], and the patient was assessed as meeting the 2004-HLH diagnostic criteria [12]: recurrent fever, cytopaenia (involving both peripheral blood lines), splenomegaly, ferritin > 40,000 ng/ml, and elevated triglycerides. On the basis of the diagnosis of *Talaromyces marneffei* infection as well as secondary HLH, the patient was given amphotericin B [13] and appropriate hormones (dexamethasone 7.5 mg q12h). However, his clinical condition further deteriorated, and he died of multiple-organ failure despite antibiotic adjustment, blood filtration, ventilator-assisted ventilation and other supportive treatments. Figure 1C shows the diagnosis and treatment of this patient in the hospital.

## Discussion and conclusions

*Talaromyces marneffei* infection is an endemic disease that is prevalent mainly in South and Southeast Asia. With the floating population, the disease has spread far beyond traditional endemic regions. Travel-related infections are being increasingly recognized in nonendemic regions such as the United Kingdom, the USA, Australia, Belgium, France, Germany, Japan, Sweden and Switzerland [6]. The areas with the highest prevalence of *Talaromyces marneffei* in China are in Guangdong and Guangxi provinces, and other provinces and cities across the country have also reported cases of infection as the floating population number increases annually. Infection by *Talaromyces marneffei* remains a major complication in HIV-infected and other immunodeficient patients. A CD4 + T lymphocyte count < 100 cells/µL is a high risk factor for *Talaromyces marneffei* infection in epidemic areas in HIV-infected patients [14]. In HIV-negative populations with impaired cellular immunity, such as autoimmune diseases, tumours, solid organ transplantation, and new targeted therapy, the proportion of *Talaromyces marneffei* infection is also gradually increasing [8, 15–18]. This patient was from Shandong Province, North China, and neither he nor his family members had travelled to the endemic area or had a history of exposure to bamboo rats. His HIV test was negative. To determine the presence of immune deficiency, anti-IFN-γ antibodies were assessed, but the results were negative. There was no history of high-dose hormone or immunosuppressant use and no basis for the diagnosis of autoimmune disease. For this patient, lymphocyte subset analysis revealed significantly decreased absolute numbers of T lymphocytes (177/µL) and NK cells (20/µL), and the CD4 + T lymphocyte count was 87 cells/µl. Whether there was other potential immunodeficiencies was not clear.

Positive fungal culture in tissue or body fluid is the gold standard for diagnosis of *Talaromyces marneffei* infection. Bone marrow sample culture is the most sensitive, followed by skin biopsy and blood sample [19]. However, due to the long culture period, usually 3–14 days, early diagnosis is difficult. The 1-3-β-D glucan and galactomannan tests can be used for preliminary screening of *Talaromyces marneffei*, but the specificity of these tests is poor [20]. Histopathology and polymerase chain reaction (PCR) also have diagnostic value for *Talaromyces marneffei* infection [21]. In this case, the patient had a long disease course and had previously tested negative by culture. When the blood culture in our hospital was initially positive, it was considered Penicillium, and the possibility of contamination was considered high，which did not attract sufficient attention. The final diagnosis was confirmed by mass spectrometry, typical culture medium morphology, bone marrow culture and peripheral blood mNGS. The clinical manifestations of *Talaromyces marneffei* infection are not specific, and diagnosis is likely to be delayed for patients in nonendemic areas without a clear history of travel, resulting in increased mortality [22].

**Fig. 2:**
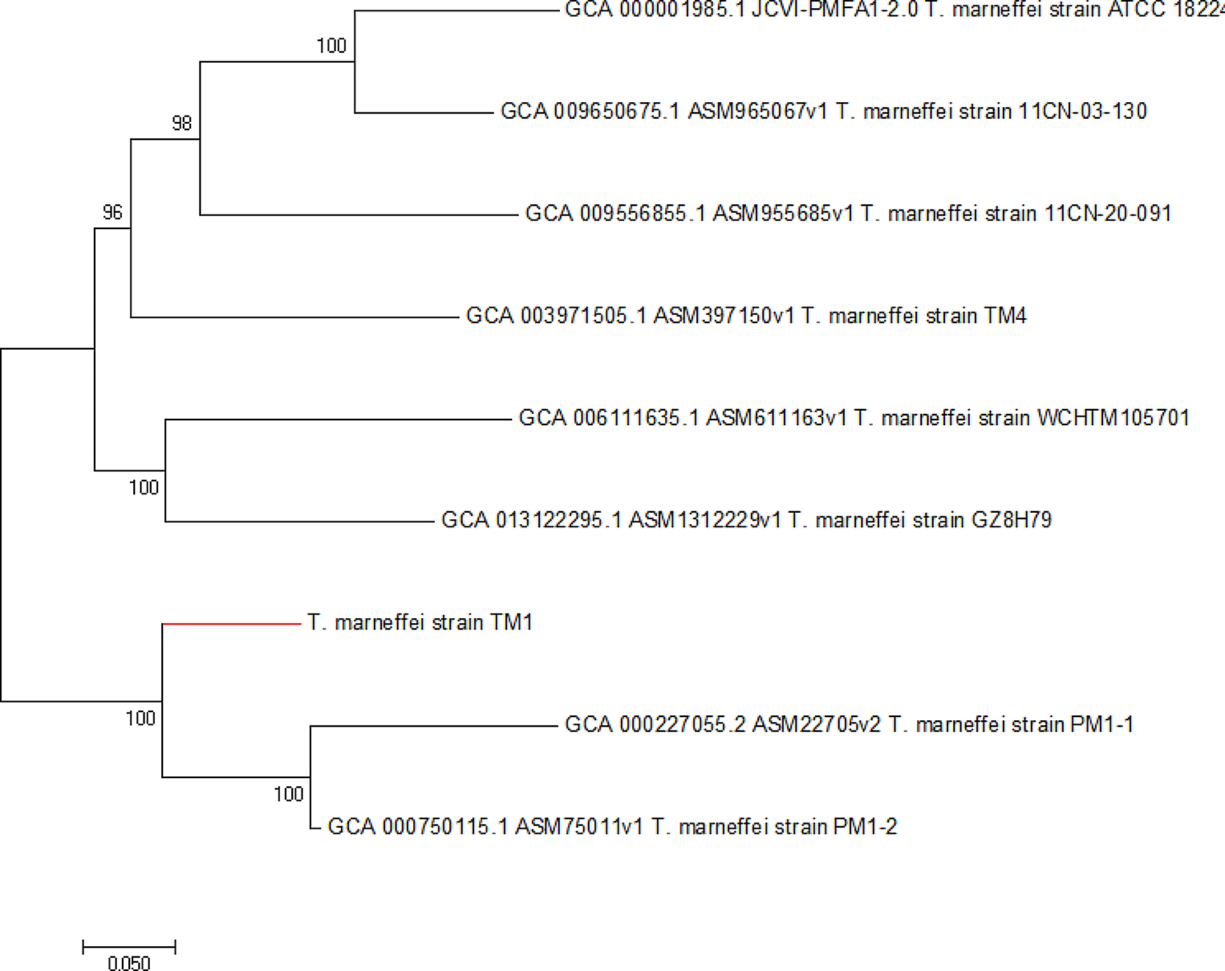
Phylogenetic relatedness of 9 *Talaromyces marneffei*. The phylogenetic tree of *Talaromyces marneffei* isolates from TM1 (with red line) and other unrelated isolates from NCBI (with black line). Nine *Talaromyces marneffei* strains were classified into two clusters, and TM1 strain was grouped with two strains PM1-1 and PM1-2 isolated from Hong Kong, suggesting a close genetic relationship between them. The numbers at each node indicate the bootstrap support from 1000 replicates

The source of this pathogen was unknown. We sequenced the isolated strain of *Talaromyces marneffei*. The DNA of the isolated *Talaromyces marneffei* strain TM1 collected from Sabaurauds Agar (SAB) was extracted using a Genomic DNA Purification Kit (Promega, USA). A DNA library was prepared, quantified and subsequently sequenced using the Illumina HiSeq platform (Illumina, San Diego, CA, USA). Raw data in Fastq format were obtained and qualified for assembly to obtain the genome. Moreover, 8 published *Talaromyces marneffei* genome sequences were downloaded from NCBI to construct a phylogenetic tree. The other genome accession numbers are shown in the Table 1. The results showed that TM1 is closely related to two strains from Hong Kong (PM1-1 and PM 1-2) because they grouped in the same cluster according to phylogenetic relatedness (Fig. 2). However, further traceability analysis of the isolate could not be performed due to the small number of *Talaromyces marneffei* genomes in the NCBI genome database and our resources. Further epidemiological investigation revealed that the patient had no history of travel to Hong Kong; however, there may be other intermediate transmission routes.

**Table 1:** 9 strains of *Talaromyces marneffei* for phylogenetic analysis

Strain	Accession number (assembly ID)	Location	Year
PM1-1	GCA_000227055.2 (ASM22705v2)	Hong Kong, China	2011
PM1-2	GCA_000750115.1 (ASM75011v1)	Hong Kong, China	2014
TM4	GCA_003971505.1 (ASM397150v1)	Guangxi, China	2018
WCHTM105701	GCA_006111635.1 (ASM611163v1)	Sichuan, China	2019
ATCC_18224	GCF_000001985.1 (JCVI-PMFA1-2.0)	Unknown	2008
11CN-20-091	GCA_009556855.1 (ASM955685v1)	Viet Nam	2019
11CN-03-130	GCA_009650675.1 (ASM965067v1)	Viet Nam	2019
GZ8H79	GCA_013122295.1 (ASM1312229v1)	Guangzhou, China	2020
TM1		Shandong, China	2022

At present, amphotericin B is still the first-line treatment option for *Talaromyces marneffei* infection. Regardless of the severity of the condition, sequential therapy comprising amphotericin B induction therapy and itraconazole consolidation therapy is recommended. Voriconazole may be selected for patients who cannot tolerate amphotericin B through induction therapy [13, 23]. During the hospitalization of this patient, liposomal amphotericin B was not available in China, and amphotericin B deoxycholate was given initially, and the dosage was gradually increased. However, due to the worsening of the patient’s condition and the rapid deterioration of renal function, amphotericin B was ultimately discontinued, after which the therapy was switched to oral antifungal treatment with voriconazole. The patient had multiple-organ failure. Even after active treatment, the adverse outcome could not be reversed, and the patient ultimately died.

With climate change and population mobility, many endemic mycoses are no longer confined to common areas, and vigilance about “new” infections is needed. *Talaromyces marneffei* disease has rarely been reported in northern China. The nonspecific and atypical clinical presentation often adds to the difficulty of early diagnosis, resulting in diagnostic delay and increased mortality, especially in nonendemic areas. We should be wary of this new infection. We hope to increase awareness of *Talaromyces marneffei* disease through the diagnosis and treatment of this patient.

## Data Availability

Not applicable.

## Abbreviations

CRP: C-reactive protein
PCT: procalcitonin
CT: computed tomography
HLH: haemophagocytic lymphohistiocytosis
TM: *Talaromyces marneffei*
